# Some Aspects of Medical Education

**Published:** 1904-07

**Authors:** John H. Musser

**Affiliations:** Philadelphia, Pa.


### SOME ASPECTS OF MEDICAL EDUCATION.*

BY JOHN H. MUSSER, M.D.,

PHILADELPHIA, PA.

It is no trifling occasion that draws together from the uttermost parts of the country one-fifth of an organization of a profession that is taxed to its utmost in the prosecution of its duties. It is not with sounding trumpets and tinkling cymbals that from the sorrowing bedside—whether in the hovel of the poor or the palace of the rich—from the excitement of the operating-room, from the stimulus of the hospital ward or the quiet but inspiring haunts of the laboratory this assembly gathers. That it is possible is a handsome tribute to the noble animus in which are embodied a spirit of inquiry, a thirst for knowledge, a longing for inspiration, a desire to uplift and to be uplifted that pervades the profession. The event is one that should be fraught with the dignity, the inspiration, one might almost say solemnity, befitting a symphonal march. For such animus, fellow members, we have just cause for self-congratulation.

It scarcely need be reiterated here that the spirit which pervades such a meeting is the outcome of that evolution which began with the introduction of precision in medicine. Just as soon as the art of medicine had for its foundation the science of medicine, the possibility of harmonious medical organization arose. When "rule of thumb" or intuition gave way to precision; idealism to realism; when speculation was displaced by fact; when, in short, inductive methods were employed and honest inquiry became the guiding star of action, then harmony could sit in council.

With medical, as with all organizations made up of human elements, it would be only human if not pervaded by the essence of science—truth. Hence with the latter wanting, jealousies and ambitions, self and selfishness would prevail to the end that the true object of organization—a fraternal spirit and conscientious seeking

*President's Address at the Fifty-fifth Annual Session of the American Medical Association at Atlantic City, June 7-10, 1904. Courtesy of the Journal of the American Medical Association.

for truth—would be as a flickering flame. A little observation will show that that society, whether county, state or national, that does not have this true reason for its existence, for its guide of action, will be a failure. The reverse is also true that that organization which may be called unsuccessful and unhappy, of snarling men and backbiting colleagues, can be labeled at once as without its true vocation, to the discredit of its members.

The great heights to which our organization is rising rapidly precludes the activities of such as have self for their basis of action, so that each day we see more and more a noble rivalry of its members to do the utmost and the best for the organization; a healthful vying of one another for its welfare. Self and selfishness are looked at askance; partisanship and faction, save for a common end—the welfare of the Association—are memories.

But, fellow members of the Association, at this the fifty-fifth session of our Association we have other causes for congratulation and felicitation. We are on the eve of a union of the all members of the profession in one harmonious organization, each individual of which is willing and proud to consider his fellow a gentleman until he is proved not to be, and not, as of yore, a knave until he is proved to be a saint. We are reaping the rewards of that untiring committee which gave to us this organization with which we now labor. We are prospering beyond measure and gathering a force scarcely to be estimated, in great part as a result of the untiring efforts of the apostle of organization—our McCormack. To say that thirty-four States have adopted the uniform plan of organization, and "that membership in the reorganized States has increased 400 per cent. and enthusiasm," writes our coadjutor, "tenfold more," is enough. We are standing out in our own manliness with the majesty that belongs thereto because of the sentiments expressed when our broadly inspired committee voiced the unanimity of feeling which belonged to the Association, in our principles of ethics. We are letting our light shine through the instrumentality of that genius of medical editors—our Simmons. From five thousand to twenty thousand in less than a decade is a statement of circulation which appeals and at the same time arouses a feeling of pride and stimulates to further effort, increased all the more when we reflect that the scientific value of the articles in *The Journal of the American Medical Association* are surpassed by none of its contemporaries ; its tone is emulated by all.

Such causes for congratulation bring to this Association greater responsibilities and weightier problems for solution. It is held to be the bounds of this address to indicate responsibilities, some trite and apparently trifling, others most weighty ; to point out duties, many so grave and vast, beyond the powers of your official to justly define.

NECROLOGY.

Before passing to the task let us pause a moment and with due reverence take cognizance of our colleagues who have since our last meeting gone to the great majority. The Silent Reaper has been as rapacious as in the past, and more than fifteen hundred fell in his swath. He has spared neither the humble nor the great. The less conspicuous but just as potent laborers in the vineyard can say with the others : "I have fought a good fight, I have finished my course, I have kept the faith."

Of those who have been honored by this Association in the past we lost two ex-presidents, the hearty, rugged, yet withal gentle and kindly Donald MacLean, surgeon and teacher, and the urbane James Farquhar Hibbard, a revered general practitioner and teacher; two ex-vice-presidents in John Bates Johnson, one of the founders of the Association, a great practitioner, an honored teacher, and Isaac Newton Love, genial and magnetic. From the ranks we lost Edmund Andrews, scientist, pioneer in surgery and medical education in the West ; George Julius Engelmann, obstetrician, scientist, litterateur, teacher, honored at home and abroad by membership in great organizations ; James McFadden Gaston, pioneer in the surgical treatment of affections of the gall-bladder and gall-ducts, an esteemed practitioner ; Franklin Staples, Hamilton Atchison West, Orpheus Everts, each obtained prominence and respect by force of character and natural attainments; Emile A. de Schweinitz, scientist and brilliant investigator, laboring not for his own renown but for that of his profession, passing away at the threshold of a most promising career ; Frank Savary Pearce, untiring and enthusiastic, who had been expected to counsel with us this week.

HOUSE OF DELEGATES.

The conduct of business under the new constitution bodes success. The two years of trial have indicated that to the House of Delegates the greatest responsibilities fall. When its members consider the great power the American Medical Association will have for weal or woe in the future and that the great body gives that power almost unstintingly to the delegates, they should justly realize the honor and confidence bestowed on them. With such realization will come honest purpose and deliberate action. All the county and State organizations must appreciate the vast duties to be undertaken and the breadth of view, deliberation and wisdom essential to their correct performance, and consequently select from their number the best of their possessions. Let us as an organization see to it that honor and homage come to those who labor unceasingly in the House of Delegates.

NATIONAL LIFE.

It can be readily seen that an organization of the magnitude and with the object and the spirit of our Association must in justice to itself and duty to its country take part in its social and political life. In order that we can be in touch with such life it would be well for the Association to devote some time to the discussion of broad general topics. We have provided for discussion at this meeting, the advisability of which may be termed tentative. We feel they can be productive of great good, and if the experience on this occasion is the least encouraging, we would urge their continuance. We had hoped to have government officials take up matters pertaining to the relation of the medical profession to the executive, legislative and administrative departments of the government. The exigencies of politics forbade our obtaining the services of those worthy to address this body. All men of political affairs are too busy at this juncture. At other times we may learn with profit from our rulers and lawmakers. As arranged for this week, so in various parts of the country in which the meetings are held, broad topics of general interest, perhaps with local flavor, could be discussed. From those in the far West we might learn much of climate or how to conquer the plague or the medical aspect of "the yellow peril." The good judgment of the committee, or the President, can be relied on to select topics suitable to the occasion.

It is needless to refer to the demands that will be made on us by the nation in the future. You will learn through the representatives of the army, the navy and the marine-hospital service what is expected of us. My distinguished predecessors, Reed, Keen and others, have pointed out our possibilities for good in the legislative halls of county, state and nation. We must have care never to be partisan or factional toward the weighty matters we will have to decide. We must realize that the power we have may be our undoing. We must realize that we are entering on dangerous ground, and that it would better become us to be bidden rather than to intrude ourselves into such territory. Indeed, one may tremble not a little at the possibilities of our development in the wrong direction. So long, however, as we hold true to the spirit which comes out of devotion to science we need not fear.

THE SECTIONS.

The work in most of the sections the past few years bears favorable comparison with that of coordinated organizations throughout the country. To inflict discussion concerning them on this general meeting is, we realize, somewhat of a hardship. Your indulgence is craved, however, for through your medium alone can the sections be reached, while peradventure the work of these sections is fully as important as that of any branch of our Association work. If you will look into it you will find the rise of the Association came with the development of section work. To enhance the value of such work is, therefore, to uplift the organization. The present methods are worthy of trial for a few years just as we should not make haste in changing the new constitution. Regarding it, may we suggest thought along these lines?

1. The continuance of the secretary for a term of years.

2. The collaboration of sections so that one topic can be exhaustively considered at the meeting of the Association without repetition in other sections. For this purpose the officers of the sections should meet for consultation six or eight months before the annual meeting. We feel we did good work by having our distinguished chairman and secretaries come together last October.

3. The division of the Association into two main sections for work in the mornings, that of (*a*) *medicine*, including pathology, diseases of children, nervous and mental diseases, materia medica, stomatology, cutaneous medicine and sanitary science; (*b*) *surgery*, including obstetrics, gynecology and the more closely related surgical specialties. The members interested in diseases of the eye or nose or throat could contribute to either section the result of any studies related to the respective section. The afternoon could be occupied by the specialized labor of the twelve sections now in existence.

Perhaps it might be thought better to have one morning, at the direction of the Section on Practice of Medicine, devoted to the discussion of general topics related to medicine, in which the entire Association could take part; another morning, at the direction of its secretary, to surgery; a third to the specialties; a fourth to sanitary science. The object is to secure the views of physician, surgeon and specialist on general topics. To instance a subject, as endarteritis, is to call up at once the possibilities for the internist, the surgeon and the specialist.

4. The sections should take up and organize special international congresses when the time is ripe for such meetings. Thus an International Congress of Dermatology could be managed by the correlated section of this Association. With the patronage of the American Medical Association any congress that could command such special energies and broad general support could not fail.

5. The section should name to the President a list of its members from which he could select, when asked, delegates to important national meetings or international congresses.

### SANITARY SCIENCE.

The Section on Hygiene and Sanitary Science should receive greater support from the organization. It is the section which must bring us in closer touch with our national life. Through it and those devoted to it we ought to secure greater respect from the body politic, and hence more wholesome power for the welfare of the community. Through it the profession should learn to take greater interest in the work of sanitary officers throughout the country. The valuable address of Billings on "The Relation of Medical Science to Commerce," and that of Thompson on "The Economic Value of Medical Science" indicate the opportunities and responsibilities of the profession. The time has gone by when sanitary positions should be used for temporary sources of income and obtained by political means. Such offices are growing more and more vital to the public welfare. They have not been attractive because the rewards have not been commensurate with the responsibility and the labor demanded. They should, we will admit, if looked at from the proper viewpoint, grant large compensation and the occupant should be guaranteed tenure of office not subject to party exigencies but dependent on value received. The fault is partly ours that such is not the case. In but few medical schools is there a serious attempt to educate sanitarians, consequently the physicians who engage in sanitary science in an amateurish way do not give a *quid pro quo*. It does not pay a community to hire them. In one of the large cities of this country it has been impossible for the health authorities to get men who were competent to take up health matters, even though the city was prepared to pay fair salaries. Speculation need not run riot in estimating what would occur in a few years if our universities would give sound, practical courses of a broad character, including some engineering, some economics and other features which would round out the man. In preliminary and subsequently in the elective studies the student should not be tempted to go into the special lines of practice. He should have opportunity to go in for sanitary science and perhaps for government service in the army, the navy, or other departments. The communities would soon find the worth of men thus educated, and the demand, we would venture to predict, would outrun the supply. In proof of the former, we may cite the fine tribute given to Wende, of Buffalo, by the citizens, *en masse* and of one accord, for the work of self-sacrifice, of unselfishness and of worth as official of the health department. Our Association could as a body and in its constituent parts encourage the medical schools to make such training possible ; they could educate the community to demand such services and insist that good compensation is to be allotted for it. The Section on Sanitary Science could take up this as one feature of its work.

But for a more grievous reason, the profession's attitude, there is public disrespect. Survival of the old dictum that a patient's woes are confidential supports too often the attending physician in failing to realize his responsibility to the community while attending to the welfare of his client. For this, as well as too often for personal reasons, he readily connives with the patient to thwart civic authority, thereby endangering a community. Instead of aiding in securing respect for and obedience to the rules of his sanitary colleague he joins in opposing him. Candor must compel us to admit that too often he fears the advent of the health official may mean some disclosure of his own incapacity. It is fair to say the official on his part is often too ready to give advice, to sneer at diagnosis or rail at treatment. To inspire, therefore, wholesome respect toward these coworkers and establish an *esprit de corps* should be one of the educational prerogatives of this section. Were we to be called to account for duties undone, the sin of not fulfilling our obligation to the community in sanitary matters would doubtless be found the most conspicuous, the one most detrimental to its interest.

Again, if not too insistent, could not this section further educational exhibits in sanitary matters not unlike the superb exhibit last winter in Baltimore, conceived and conducted in the main by our present official of the Sanitary Section? The Association could not do better than issue a grant for the furtherance of this object.

### THE SCIENTIFIC EXHIBIT.

It is gratifying to see the interest and success attendant on this new feature of section work. It is another atribute to the belief in the permanent tenure of office of an official when we have the good fortune to get a good one. Attention is particularly called to this exhibit to ask the Association and the other sections to look to the conduct of somewhat similar exhibits. The Section on Ophthalmology showed the possibilities at the time of the Helmholtz anniversaries. When a section takes up a subject let us not have the clinical, pathological and therapeutical aspects alone discussed, but bibliographical and historical features brought out. Let such features be as national as possible. What a fine exhibition it would be to have in connection with, say typhoid fever, a loan collection of the pictures of authors and investigators, of the various editions of their works and of the material things with which they worked, illustrating the evolution of their knowledge of the subject. Who would not be thrilled by a picture of the wards of the old Pennsylvania Hospital where Gerhard worked, of the instruments or materials he worked with, how he lived and what he did; of the first edition of his works, indeed of the manuscript of them? We must confess to a quiver of delight, mingled with reverence, when permitted to handle the manuscript on which Stillé inscribed his communication to the Paris Society. This is not the time or place to indulge in a homily on the value of such exhibits, agreeable though it might be. Through them, as ot biography and sculpture and painting, we keep in touch with our history. Foster efforts in these directions, gentlemen of the Association, grant liberally to sections their small demands on you, to the end that you will be stimulating the best instincts in our profession.

### THE ASSOCIATION OF LIBRARIANS.

We are not far amiss when we call attention to an organization which in a quiet, unobtrusive way is bringing together the members of the profession on a common ground, where tares do not spring up and thistles do not blossom, but instead in an atmosphere clear, refreshing, invigorating. Jealousies do not thrive in libraries; books soften ambitions. No stronger, more liberal friendships can develop than those that come to book-lovers. We owe a debt of gratitude to the Library Association and to its distinguished president, under whose fostering care the influence and power of the Association are unfolding as the hardy rose, exhaling like it a fragrance which cheers and stimulates. Of the many abundant works we owe him, none, we venture, will be more satisfying to him and more lasting for good than the work of the Association of Librarians. This Association should hold not its hand nor stint its heart in upholding its educational efforts.

THE RUSH MONUMENT.

Before this session adjourns, by proper ceremony, graced by the presence of the President of the United States, with an oration befitting the occasion by our distingushed colleague, Dr. Wilson, the monument erected by the Association to the memory of Benjamin Rush will be dedicated. The Association is to be congratulated on the completion of this tribute in perpetual bronze to one of the most distinguished of our countrymen. Not alone should we rejoice on the fruition of this agreeable task, but also because it augurs for the erection in the future of tributes to the memory of others full worthy of our reverence. The invigorating, stimulating influence that comes from having about us our heroes of the past in stone or bronze behooves this Association and its constituent bodies to strain every effort to encourage in this manner hero worship. It is due ourselves to perpetuate the inspirations, the thoughts, the feelings in the counterfeit presentment of our fellows. We are not of those who decry the world because it has not recognized our Jenner, our Pasteur, our innumerable heroes either by reward in their lifetime or by permanent tribute. We do not begrudge the meed of praise and the plenteous bounty which flow to our warriors and statesmen. It is true, their glory has been attained often by carnage, lives sacrificed to avenge some insult. It was not life that was at stake. It was principle or thought or feeling or, our misanthrope suggests, greed. What countless lives are sacrified for aspiration; what thousands of innocents for national gain! So, to the actors who stand for such, unstinted regard is given. The public neither appreciates the loss nor the saving of life. Hence, so long as it does not come close to them, civic murders from bad hygienic conditions do not move them. Even when home is devastated they submit to that which they consider mystifying or providential, and merely go their way. They are callous beyond measure to the presence of epidemics. They do not consider for one moment the power they have to assuage the virulence or even wipe out the existence of pestilence. Every effort for amelioration, as we see in tuberculosis, comes from our profession. With such lack of appreciation of their own welfare we need not expect much appreciation from them of our efforts.

In all honesty, we must admit among ourselves, we may not deserve public appreciation. If we realize the truth we may make amends. Could the public be expected to revere and respect when, as usual in the past, perhaps too common now, the profession was divided against itself, not from principle but generally because of pocket, of sordid ambition, of devouring jealousy? Look ye to your county societies! Do the brethren dwell together in harmony? Go to the warfaring court-rooms! Do we emerge from them respected and respectful of one another? Until such sores are healed as they happily will be when science—and hence liberality of mind and largeness of heart—is furthered by associations like ours, it must remain to us a cherished heritage and privilege to worship our heroes alone. When we learn to respect ourselves, then will we be respected.

MEDICAL EDUCATION.

The object of this Association shall be for the purpose of elevating the standard of Medical Education.

This Association has been, should be, and we trust will be the storm-center of legislation for reform in medical education. Since the memorable editorials of Wood in the old *Philadelphia Times*, and the masterly papers and addresses of Pepper and the practical action of the University of Pennsylvania there has been virile progress. In most respects it seems definitely settled as to the course of education a candidate for the degree of medicine should take. Questions of pedagogy are still debatable, but we take it that that student who wishes the quickest returns, the most lasting remuneration, perennial stimulation of the intellect and continuous enjoyment in the pursuit of his labors, should take a college education of three or four years, a four years' course in medicine, and if possible, a hospital interneship.

Reference need only be made to the reports to this Association, to the famous report of the majority committee of the Association of American Medical Colleges, to the numbers of the *Journal of the American Medical Association* comprehensively devoted to education, and to many recent admirable addresses in support of the statements.

There is talk about maximum and minimum requirements, about the laboratory and hospital courses, the merits of didactic and clinical teaching—a mass of material brought forth from the viewpoint of the educator, or looking to the welfare of the medical profession. We do not minimize the value of such lines of discussion. It has brought us to the position we have attained. But what of the medical student? Should we not look at education from his point of view? Is he quite able to decide whether he should take up the profession of medicine? We hold that a great duty is due the aspirant for medical honors from teacher and practitioner. It is a kindness due him to point out the best methods of securing such education as will yield him results commensurate with the time and expense required. It would be a greater kindness to be enabled to show him that by reason of intellectual temperament or of physical or moral qualities he is not likely to reap the rewards he is anticipating.

The large majority of medical students do not have a good reason for studying medicine. They are ignorant of the mental and physical demands made on them. They are attracted by an uncertain glamour and a spacious glory, and heedlessly they go in. The failure of a large percentage of graduates in medicine to acquire more than a bare existence, and too often not even that, proves that they were not educated properly, not fitted temperamentally or physically, to pursue its duties. Should they not have opportunity for learning of the responsibilities and difficulties, rather than to have the brighter phases glorified? Would it not be well to have in our college curriculum a course of lectures for the student who contemplates entering a profession, pointing out the rocks and shoals, in his prospective career? An eminent practitioner, not connected with medical schools, would light up and darken the pathway in due proportions. Then, too, should not, as in the Army and Navy, some physical tests be required? The trophy is to the robust, and sad will be the career of the man who is physically handicapped.

If there were any doubt about the value of a college degree to a man entering the medical profession it could be set at naught since the report of the Mosely Education Commission. Quotations like the following, while not pertaining to medicine alone, the result of extensive inquiry and mature deliberation, supported by the statistics they give, uphold the contention of a large employer, that "for 99 per cent. of the non-university men, it is hopeless to expect to get to the top." One opinion they express is that "there is still room for the boy of marked ability 'to come through,' but that his difficulties are greatly increasing, and that, useful as he is, his usefulness would have been greatly enhanced had he had the benefit of a college training." Still another commissioner reports that while only "one per cent. of the entire population of America has received a higher education in her colleges and universities, this one per cent. holds more than forty per cent. of all positions of confidence, of trust and profit." It is well known that the "geist" of the individual brings success, for which they say "it is recognized that the educated man takes in a wide horizon and puts more 'soul' into his work."

The essential of success in any department is diagnosis, which requires powers of intellectual penetration and discrimination. President Thwing has again forcefully urged that "the reasoning of the mathematics—and mathematics is only reasoning—tends to promote *clearness* and *accuracy in perception*, *inevitableness in inference*, a sense of logical *orderliness*. The study of the languages represents the element of *interpretation*. The study of history means the interpretation of life." Are these not the main studies of a college education? While they may promote scholarship, they surely cultivate thought. It need scarcely be pointed out to this audience that to be a thinker is the salvation of the physician.

To the plea that the acquirement of a college degree takes up too much time and requires too much money, the material answer can be given from other sources to the effect that "the men whom you are surprised to find holding such important positions in factories, though not much over thirty years, are the very men who did not leave the technical college till they were twenty-three or twenty-four; the graduate may have been twenty-five before he donned a jumper, but in five years he learned more with the college training he had as a foundation than the regular journeyman of fifteen years of actual work in the shop." The experience of teachers who have watched the alumni agrees in that the college graduates get quick returns and soon acquire a position of independence.

The poor boy, therefore, need not be deterred, for if he has the spirit and energy to work his way through four years, two years or three years more will be but very little in the final summing up. If the student only knew that the purchase of the best education, whether reckoned in time or money, was the most economical investment, in that as to the former, a thorough education at first is time-saving in later years, and as to the latter, the money outlay is returned more quickly, in more immediate work and larger pay.

There should be one educational requirement—the equivalent of that for which a first-class college degree stands, whether received at a high school or university.

After entering the medical school with, it is presumed, the proper educational attainments, his career the first year should be closely watched. The school has too many students if it does not have enough instructors in the first year to be able to judge with a reasonable degree of accuracy of the character and moral stability of the men. This is not to be taken in a prudish sense or with too critical a scrutiny of habits which are the overflow of the animal spirits or the expiring exuberance of the boy approaching manhood. This can be said that a student who does not play fair in his exercises, who cheats in one demonstration or evades another, who does not show manliness, frankness and truthfulness in his first-year duties, will not be a good diagnostician. He will cheat himself; he will cheat his patient. The teachers of the first year, or at least the second, should know this and block the student there and then. It would be a kindness. Let us then agitate whether we should not have a certificate of manliness, a certificate of health as well as a certificate of mental proficiency, before we admit students to our medical schools or permit them to go beyond the first year. Let us not be decoys, alluring them on to later destruction, but rather be guardians, wrapping the strong arm of experience about them to lead them to the fitting pathway.

Having permitted the student to pass further in his pursuits, we still owe him much. We must see to it that such course is given him the first two years of his student career that he will acquire such fondness for the science of medicine, such reverence for the exploration of its truths, that until his dying day devotion to it will be his stimulus and solace. As a corollary, we must insist that medical schools secure the best men in the market for these places and pay them salaries commensurate with their ability—good living salaries.

It is in the first and second years of his career that the foundations are laid whereby the student becomes the medical thinker. To quote again: "The power of thinking should not be of a base and barren character. The thinking should represent and be concerned with a fine and rich content of knowledge. It should have the exactness of intellectual discrimination; it should have the fulness of noble scholarship; it should embody a culture which is at once emotional and esthetic and ethical, as well as intellectual."

That a desire to relieve suffering, to extend sympathy, to save life, is the impulse of the physician, we all admit, but where is the man among us who will not also admit that a scientific habit more quickly brings it about and more surely sustains and fortifies the humane instinct through the trials and tribulations of exacting practice? That prosecution of professional duties soon becomes commercial that does not have for its basis a true spirit of scientific inquiry. How miserable must that life be which conducts an exacting, drudging, daily routine with only material reward in view. Few are the practitioners who have this sordid view; we can be as sure medicine would soon be forsaken if this view-point alone were considered. Hence in the laboratory of the first two years must be aroused and fostered the stimulus for lifework.

The final years should be clinical years, and the last should be in a hospital. The medical school that allows its students to think such opportunity is not due them is most unfair to them. As our schools are now constituted, most of them can not give such requirements. The students should know, however, such requirement is necessary. What has been said regarding the preliminary college education applies equally forcefully to the hospital training. He is thrice armed who enters the arena thus equipped. Medical schools that can not give such education are cruelly unkind and unjust to the students by having them think it is not essential. Medical colleges that pass off a hospital training for one that is not truly such fake their students. The student who pays well for his training has the right to demand such as to fit him for immediate action.

It is not the fault of the medical school alone that he can not get it. The public that cries out when there is mistake in diagnosis, fault in treatment, and that shakes its head at the deficient education of our students, must share the blame with the medical school. The public admits that its individual members may at any moment almost be at the mercy of a half-educated physician. It is not necessary to recount, for it is well known, how on land or sea, by day or night, some event may arise in an individual life, the care of which may mean life or death. Even with this knowledge they withhold means to relieve themselves. They admit the necessity of a hospital training. But they, and particularly the public in control of hospitals not used for teaching, say each medical college should have its teaching hospital. They do not appreciate that to give an education which involves a hospital course would require an expenditure of $500 a year for four years by each student. It has been estimated that the cost of maintaining a plant and paying salaries sufficiently large to accommodate 600 students would require the above outlay by each student. Unfortunately, it is impossible to expect students to pay such figures, as it would render entrance into the profession almost prohibitive. It is manifestly impossible, as medical schools are constituted now, to educate all the students of the land properly. Hospital training can not be given except by a few favored institutions, because the doors of hospitals are closed either by the governing body of the hospital or by the teachers in the medical schools.

We believe, personally, if a decree should be issued that no medical school, including its hospital, should exist except on the fees derived from students, but little hardship would follow. The lessened supply of students would increase the demands on the practitioner, so that larger returns would follow. The poor student would sacrifice and strive to get a degree, knowing then he had a good asset. A diminution in number and an increase in quality is demanded alike by the public and the profession. Such diminution in number would mean that the student would get back his investment quicker and in larger amount than at present, hence good men would be attracted. If we could abolish sentiment for sense and educate accordingly, there might be betterment all around. As it is now, medical students receive part of their education through the bounty of the State or the charity of the public, as such education can only be given in endowed institutions. The public is taxed so that the prospective physician can make a living. Is it right that it should be? Perhaps a mechanic should demand such right to make his son a good workman. We must all admit it is the duty of the State to educate the youth, so that good citizenship is maintained; we can question whether the State should educate the members to obtain a livelihood.

With the same indifference that the public views an epidemic's march they allow hospitals that are engaged in teaching to suffer for the want of funds. Moreover, they close their doors to the advent of teaching in the hospitals under their control. We must admit those who do not appreciate the true function of a hospital have some ground for their contention. Ruled by sentiment chiefly, unfortunately an impracticable master, they sympathize with the patient who still harbors the belief of old that the medical student is one of a class that prowl about not unlike harpies. The public does not realize the difference in the student of today and the student of tradition. We can not hold to account the governing body of the hospital who has the point of view that it is harmful to a sick person to have them under the surveillance of an alleged student rabble. We must admit some patients become alarmed, particularly in institutions where they know they will have the sympathy of the governing body. An analysis of motive will show that the usual patient who will not allow a judicious amount of clinical demonstration when the sense of delicacy is not offended, is truly selfish, in that there is prevented that increase of knowledge and development of skill whereby suffering of others may be alleviated. A little encouragement from the officials would allay alarm on the part of the patient. The desire to help others is infectious, and when one yields in a word, others vie in the work.

The truth of the matter is that in hospitals in which teaching is carried on, rarely, if ever, do the patients complain. Indeed, it is the experience of those teaching institutions that judiciously conducted instruction is appreciated by the patient. In one hospital we might name most of the inmates are pay patients, giving $7 a week willingly, because they know they are buying the services of the best practitioners in the land—the teachers of medicine—which service they could not get at tenfold the figure. The fact that teaching hospitals are overcrowded, not by the poor alone, but by people independent of charity, shows that clinical instruction is not a bugbear. If the governing boards would know that while a few patients might be alarmed, on the whole most of them would be gratified by the attention paid them, and their sense of rectitude and manliness appealed to by the satisfaction that they are doing some good in enlightening students, so that others could be relieved; that their administration would be stimulated to do work beyond criticism; that the nurses would be aroused to better activity while under the observation of those not connected with the hospital; that the internes would do their very best to have most complete studies of the case, and finally, that the chief in attendance, compelled to do his best at the risk of his reputation, they would gladly open their doors, even at the discomfort of the few, but to the advantage of the many. In short, the hospital should have teaching not to oblige the medical school, but for its own survival and regeneration. The benefits the student derives by the object-lesson of an orderly hospital can not be estimated. Will not every member of a hospital board admit that his own character, his own sympathies, have been benefited by his connection with the hospital, even though, perhaps, he has not the advantage of an impressionable age? Can he not see therefore, how the youthful student can be influenced in thought and character and feeling? He can not lightly toss aside this responsibility, nor even hide it by putting the onus of medical education on the teaching hospital. Every dollar endowing a non-teaching hospital robs the teaching hospital which is engaged in this larger duty.

It is true a class who are compelled to have hospital attention may not sympathize with this feeling. How can we obtain the confidence of this class? Let us organize such association of prominent people in our teaching centers who will agree to have any operation, any feature of disease witnessed by medical students, at the judgment of their attending physician. It would be well if the individuals of such an organization would agree, first, to undergo hospital treatment; second, to be the object of observation by students; third, to have an autopsy performed in case of untimely end. We feel sure that it would be wholesome individually and publicly if each would agree to ward treatment. Such association would rob hospitals of their terror and teaching of its dread. No one can deny that on the whole the public would be benefited from whatsoever point of view we look at it. Indeed, the public ought to learn that disease is an enemy to themselves and their country. Just as we make sacrifices in time of national warfare, so we should be willing to make sacrifices in the daily battle for life. Just as aristocrat and plebian, landlord and tenant, fight side by side in the former, so they should array in solid phalanx in the latter.

But there are hospitals willing to admit students, and yet the privilege is not availed of. This arises because the teaching force of the medical college is not willing to sink its personality and allow the student to go wheresoever he will for his instruction. Courage and some sacrifice is required perhaps. But when one thinks of the mighty opportunity and the frightful waste, it is saddening. Every hospital should be a school. The fourth year should be so arranged that the student could avail himself of the advantages of hospitals in the immediate vicinity. Let each teaching body have the students understand what he must see and do, and trust you the true student will see it at the best place and with the best men. He must be accountable, of course, with a rigidity that means the exact acquirement of knowledge. To this end the first two years could be well spent in the properly equipped laboratory university, whether in town or country, the third in the authorized hospital of the school, the fourth in extramural hospital work.

Is it not anomalous that the hospital boards give to the nurses who are to act as aids to the physician the highest opportunities, and yet deny it to those who are to give orders to the nurses? This, of course, arises because training-schools are the product of modern thought and have not been trammeled by tradition.

But all this talk of the primary education avails but little if we do not see to it ourselves that education is continuous; that from the day of our graduation, forwards, we do naught but toil, toil, toil. It should go without saying, as a mere business proposition, that unless we unceasingly labor but little of the fruits do we pluck. It is demonstrated in a practical manner, for when we look about us and find the methods of those of our brethren whose labors are not in vain each one bears the scent of midnight oil.

The development of post-graduate schools, the growth of libraries, the groans of the printing-press, the enthusiasm of medical societies, all testify to the spirit of persistent self-education that is abroad. It is not for me to urge further the importance of each of us taking from time to time months for study and reflection. Every active doctor should have his sabbatical year. We dare say, extended observations would support the belief that the gain is one hundredfold for each dollar invested in educational outings, and for every hour thus employed, ten is added to life. To finance a medical man from first to last successfully, we dare say, spend all net earnings of the first five years on self-education; after ten years, ten per cent. of the annual net earnings for an assistant, continuing the twenty-five per cent. investment for five years. Health, happiness, increased usefulness to the community, a success, which never comes from eccentricity, equal to doubling capital every five years, would follow.

In the course of our work it is necessary for us to halt from time to time and review. Not alone must our mental storehouse be swept out, refurnished, but we must constantly "jack up," if the term is permissible, our mental and moral machinery. Few of us are there who will not find as our days grow fuller an unconscious tendency to slight our work, to become slipshod, to hurry over matters. It is partly an evidence of overwork. The post-graduate school is the salvation. No obstacle can withstand the continuity of drill, which the earnest of us keep in action. If, to continue the imposition, asked what element of character is perhaps lacking to the greatest detriment to the profession and public, we possibly, one and all, would say courage. This is seen in the hesitancy which members of the profession show in giving an opinion, in advising an operation and in asking for an autopsy. How much confidence is destroyed by the want of free, frank avowal of the physician that he does not know on the one hand, or of clear, precise statement of his judgment concerning a case on the other. The greatest success in life is confidence! How many lives are lost by the worker in internal medicine not advising early and unequivocally an operation for fear he might be wrong in his diagnosis! And how many more are lost because the surgeon lacks courage to do, either because he fears the patient may die, and his record be marred, or because he may operate when it is not necessary! We must admit we have had some operations done when they were not required, but let it be said to the credit of modern surgery, we have never seen an operation of such character performed by the right man that did any harm to the patient. On the other hand, the resort to operative procedures early, and in cases that even yet are not considered of surgical relief, has saved lives and lessened suffering to a degree that far overbalances the now and then futile measure. It may be assumed, without contradiction, that every case of bad appendicitis that happens to get well without surgical relief has in its wake three or five that die for want of an operation. In other words, the pernicious influence of a surgical case that recovers without operation is evident in creating the hope that other cases demanding operation might get well without it.

And the man who does not want an autopsy—not only the centers of courage need stimulation, but too often the entire medical storehouse needs refitting. Something best known to the physician himself, we trust, is lacking in the one who treats an obscure case for a day, six days, six weeks, and then does not want an autopsy. If true to himself, true to the demands of his profession, courage will not fail him. In our mental and physical round-up, we must see to it that courage is given a new backbone from time to time.

But, fellow members of the Association, not alone as members of this organization may we indulge in self-congratulation, but as members of a profession whose limitation knows no bounds, we may join in felicitation. Neither language, nor creed, nor country fetters our profession's munificent sway. The thoughts of Ehrlich, in Frankfurt; of our own Welch or Councilman, of Kitasato, in Japan, are correlated. The knife of Mayo in America, of Robson in London, of Kocher in Berne contributes to the relief of suffering in far Cathay. The founts of Lister's genius and Pasteur's divine inspiration bring countless blessings to England, to India, to France and to Africa. What a stimulus it is to realize that, howsoever small the contribution of the humblest of us may be, its impulse will be felt in climes near and far and ages present and remote! What awe can not but overtake us when we consider each heart throb we study entwines us to Harvey of two centuries ago; with every percussion tone reverberates the sound of Lannec's voice of a century; with each vaccine inoculation, the simple observation and reasoning of Jenner to stimulate our question and deductions!

We rejoice together and cherish our history, by the warp and woof of which we are woven to the past. What heritage for us and our children! Dead must be the soul that wearies of communion with the spirits of the past; deep must be its slumber on which falls the thought of centuries; lethargic its activities that are aroused not by the deeds of heroic men! "Honor and fortune exist to him who always recognizes the neighborhood of the great, always feels himself in the presence of high causes." We worship together our science, devotion to which brings forth character, smothers egotism, levels pretension, drives out solitude, develops such loftiness of thought which can see that "against all appearances the nature of things works for truth and right forever." Of our art, let us see to it that when the final summons comes it can be said of us, "Greater love hath no man than this; that a man lay down his life for his friends."

